# Influence of Electric Field on Proliferation Activity of Human Dermal Fibroblasts

**DOI:** 10.3390/jfb13030089

**Published:** 2022-06-29

**Authors:** Almaz Kamalov, Mikhail Shishov, Natalia Smirnova, Vera Kodolova-Chukhontseva, Irina Dobrovol’skaya, Konstantin Kolbe, Andrei Didenko, Elena Ivan’kova, Vladimir Yudin, Pierfrancesco Morganti

**Affiliations:** 1 Research Laboratory “Polymer Materials for Tissue Engineering and Transplantology”, Institute of Biomedical Systems and Biotechnology, Peter the Great St. Petersburg Polytechnic University, Polytechnicheskaya 29, 195251 St. Petersburg, Russia; shv_misha@mail.ru (M.S.); nvsmirnoff@yandex.ru (N.S.); vera_kodolova@mail.ru (V.K.-C.); zair2@mail.ru (I.D.); kkolbe@yandex.ru (K.K.); vanilin72@yandex.ru (A.D.); ivelen@mail.ru (E.I.); yudinve@gmail.com (V.Y.); 2 R&D Unit, Academy of History of Healthcare Art, Lungotevere in Sassia 3, 00186 Rome, Italy; pierfrancesco.morganti@iscd.it

**Keywords:** polyimide, graphene, fibroblasts, electrostimulation, skin

## Abstract

In this work, an electrically conductive composite based on thermoplastic polyimide and graphene was obtained and used as a bioelectrode for electrical stimulation of human dermal fibroblasts. The values of the electrical conductivity of the obtained composite films varied from 10^−15^ to 10^2^ S/m with increasing graphene content (from 0 to 5.0 wt.%). The characteristics of ionic and electronic currents flowing through the matrix with the superposition of cyclic potentials ± 100 mV were studied. The high stability of the composite was established during prolonged cycling (130 h) in an electric field with a frequency of 0.016 Hz. It was established that the composite films based on polyimide and graphene have good biocompatibility and are not toxic to fibroblast cells. It was shown that preliminary electrical stimulation increases the proliferative activity of human dermal fibroblasts in comparison with intact cells. It is revealed that an electric field with a strength E = 0.02–0.04 V/m applied to the polyimide films containing 0.5–3.0 wt.% of the graphene nanoparticles activates cellular processes (adhesion, proliferation).

## 1. Introduction

The human organism has an endogenous bioelectric system that constantly produces and receives natural electrochemical signals in various tissues, such as the nervous system, skin, muscles, heart, bones, etc. In recent decades, it has been demonstrated that exogenous electrostimulation can be used to modulate fundamental aspects of cell vital activity and behavior: adhesion, migration, proliferation, apoptosis, and differentiation. Vital functions and behavior of many types of cells can be effectively controlled by delivering electric signals through a conductive scaffold [1]. The possibility of non-invasive and reproducible cellular regulation increases the efficiency of regenerative technologies that are now being developed for a wide variety of clinical applications. 

To understand the mechanism of the influence of electrostimulation at the cell level, it is first necessary to determine whether the electric field affects a cell directly or indirectly (via changing physical or chemical factors in the extracellular medium). The electric field can act upon a cell in one of the following ways. First, it can act inside the cell and affect the profiles of motion and concentration of charged cytoplasmic molecules. Second, it can change the transmembrane potential that has an influence on membrane reaction and activates ion transport through the plasmatic membrane (which, in turn, controls cell proliferation). Third, the electric field can act along the plasmatic membrane and cause electrophoresis-assisted accumulation of surface molecules or modulate conformational states of membrane proteins, which affects the cell-substrate interaction and adhesion mechanisms [2].

In addition to reactions of the cells that are traditionally considered electroactive (nervous cells, myocardium cells, muscle cells, etc.), the influence of various electrostimulation protocols on keratinocytes, dermal fibroblasts [3], and adipose tissue mesenchymal stem cells has been described. In the last decade, advances have been made in the methods of wound treatment and skin revitalization and in tissue engineering methods that use the conductive nature of skin and sensitivity of skin cells to electric signals. Smart wound dressings, patches, and bioelectronic medical devices based on biocompatible conducting materials have proven efficient in the stimulation of the regeneration of soft tissues and in the restoration of skin homeostasis [4,5,6]. 

Advances in materials science, microelectronics, and tissue engineering facilitated the development of bioelectronic technologies, which involve the use of biocompatible polymer-based composite materials. Introducing fillers (nanoparticles of various chemical compositions, shapes, and sizes) into a polymer scaffold makes it possible to adjust the properties of the resulting nanocomposites, namely, to increase their mechanical strength and elasticity, modify surface properties, and vary the value and type of conductivity [7,8,9,10,11].

Metals and their oxides are used as additives for polymer electrically conductive composite materials: Fe_2_O_3_, TiO_2_ [12], Mg, Se [13], SnO_2_, [14], cesium bromide [15], silver [16], and gold nanoparticles [17,18]. Materials based on polymers of aliphatic structure—PVOH, PEG, chitosan, and cellulose—are used both in engineering and in medical devices. Electrically conductive polymer films are used as adsorbents [19,20], separation membranes [21], and biosensors [22].

The structural, surface, mechanical, and electrophysical properties of the composite scaffolds intended for use in tissue engineering should be close to the corresponding parameters of a living tissue, i.e., these materials should possess biomimetic properties. The scaffolds are prepared of natural (chitosan, polyhydroxybutyrate, polylactides, gelatin, alginate, fibroin) [23,24,25] and synthetic polymers (polysiloxanes and aromatic polyimides that have good processability, mechanical properties, and chemical stability). 

A conducting composite biomaterial consists of a polymer scaffold and electroactive components, mainly carbon nanoparticles [26,27], conducting polymers [28,29], or metals [30,31,32,33]. Conducting fibers, films, and membranes based on nanofibers, hydrogels, cryogels, and foams have been developed by now [34,35]. 

Application of bioelectronic devices in wound treatment is being actively researched [36]. Electrostimulation of wound tissues may reduce infection, increase cell immunity, increase perfusion of physiological liquids and medicinal preparations, and, as a result, accelerate healing of wounds of various etiologies [37]. The electric field can induce migration, proliferation, and differentiation of cells and stimulate the growth of granulation tissue and restoration of innervation and angiogenesis, which provides faster and more efficient healing of a wound [38]. 

To provide sufficient biocompatibility and biological activity, the conductivity of a material should be close to the conductivity of living tissues. Excessively high conductivity may cause cell death (when the value of current strength exceeds their survival threshold), while materials with relatively low conductivity (too resistive) may cause overheating after applying voltage. In this case, cells die due to the denaturation of proteins and formation of toxic products in a culture medium in vitro or in tissue liquids in vivo [39]. 

Polyimides (PIs) are used in medical devices as materials for bioelectrodes, cardiovascular catheters, and devices for delivery of stents and drugs [40]. A distinct feature of PI materials is the high stability of their mechanical, dielectric, and thermal properties over a wide temperature range (from cryogenic temperatures to almost +400 °C). Biological properties of polyimides depend on their chemical composition and the morphology of polyimide-based materials. To use these materials in biomedical technologies for electrical stimulation of cell processes, it is necessary to impart conductivity to polyimides. The polyimide based on 1,3-bis(3,3′,4,4′-dicarboxyphenoxy)benzene (dianhydride R) and 4,4′-bis(4- aminophenoxy)biphenyl (diamine BAPB) [41] is a promising polymer for applications in cell technologies. Conductivity can be imparted to R-BAPB by introducing carbon particles into the material.

Graphene holds a specific place among conducting carbon particles; it has an sp_2_ hybrid two-dimensional honeycomb lattice. Graphene possesses a unique combination of properties: high conductivity (charge carrier mobility up to 200,000 cm^2^ V^−1^s^−1^) [42], mechanical strength (Young’s modulus ∼1 TPa), chemical stability, and optical transparency (optical transmission coefficient ∼97.7%) [43].

Aromatic polyimide macromolecules contain cyclic fragments, between which π-bonds are formed. This type of intermolecular interaction leads to the formation of two-dimensional morphological elements. It was shown in [44,45] that graphene particles have optimal structure and properties for the formation of a macrocluster in the polyimide matrix. This leads to an increase in the strength, modulus of elasticity, and electrical conductivity of the composite material. The composites based on polyimide and graphene demonstrate increased heat conductivity (1.0 W/mK), high elastic modulus (4 GPa), hydrophilicity [46], and increased electric conductivity 94 S/m (5 vol.% Gr) [47] with predominantly electron charge carriers. Biocompatibility of graphene has been demonstrated [48]. 

The goals of the present work included the development of the composite materials based on the R-BAPB type polyimide and graphene for medical and biological applications; and studies of their biocompatibility, electrical and mechanical properties, and performing in vitro experiments for the purpose of investigating the influence of the electric field on cell vital activity. 

Dermal fibroblasts are drivers of regenerative processes that occur in damaged skin and soft tissues [49]; therefore, the culture of primary human dermal fibroblasts was selected for estimation of biocompatibility and optimization of properties of the polyimide/graphene conducting materials that may be further used in cell technologies and tissue engineering. 

## 2. Materials and Methods

**Figure 1:**
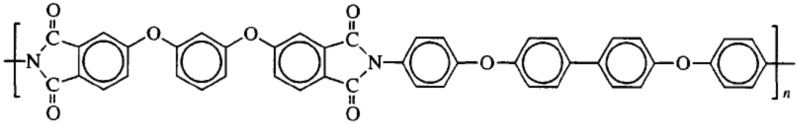
Chemical formula of R-BAPB polyimide.

R-BAPB polyimide was selected as a scaffold material for a composite film; its chemical formula is presented in Figure 1. 

The precursor of R-BAPB is the poly(amido-acid) (PAA) obtained by polycondensation of the mixture of dianhydride P and diamine BAPB in N-methylpyrrolidone (NMP) at 25 °C. PAA with molecular mass Mw ~60,000 g/mol was used in this work. The PAA solution was mixed with graphene suspension in NMP for 15 min with an ultrasonic generator (IL 10-0.63 (Ultrasonic equipment Inlab, St. Petersburg, Russia)). The frequency and power output of the generator were equal to 22 kHz and 800 W, respectively. According to the manufacturer (GRAPHENE MATERIALS, St. Petersburg, Russia), the average size of graphene flakes was 30–50 µm, and their thickness was 0.34–4.0 nm. The composite films were prepared by casting the mixture of 15 wt.% solution of PAA and graphene in NMP onto a glass support followed by drying at 60 °C for 24 h. Thermal cyclization was performed by stepwise heating; the samples were subjected to heating at each temperature (T = 100 °C, 200 °C, and 300 °C) for 1 h. Then the films were heated at 360 °C for 15 min and cooled down to room temperature at a speed of 10 °C/min.

### 2.1. Characterization of PI + Gr Composite Films

Scanning electron microscope (SEM, Supra-55 VP, Carl Zeiss, Oberkochen, Germany) was used in the studies of the fine structure of the composite films in a secondary electron mode. The investigated films were cryo-cleaved at temperature of liquid nitrogen. The fracture surfaces were fixed on the special microscope holders and sputtered by a thin layer of Pt.

The DC conductivity of films (U = 10 V) was measured at 25 °C with a Keithley 6487 picoammeter; both 2-electrode and 4-electrode configurations were used.

The AC conductivity-frequency dependences of the PI and composite films were obtained with the use of a Concept 22 broadband dielectric spectrometer (Novocontrol Technologies GmbH & Co. KG, Montabaur, Germany) equipped with an ALPHA-ANB automatic high-resolution frequency analyzer. The obtained values of dielectric permittivity and dielectric loss varied from 1 Hz to 1 MHz; the amplitude of measurement signal was 1 V.

Before measurements, platinum electrodes 10 nm thick were sprayed onto samples. The samples were placed between two parallel plate electrodes.

Mechanical properties of the samples were studied with the help of an Instron 5943 universal testing machine; the base length was 10 mm, and the sample extension rate was 10 mm/min. 

### 2.2. In Vitro Studies of Cell Behavior

The in vitro studies involved the culture of human dermal fibroblasts obtained from the Collection of cell cultures of the Institute of Cytology RAS (Saint Petersburg, Russia). The cells were cultured in DMEM nutrient medium (Paneco, Moscow, Russia) with added 1% of L-glutamine, 1% of antibiotics (100 units/mL penicillin, 100 µg/mL streptomycin), 1% of fungizone (25 µg/mL amphotericin B), and 12% of fetal bovine serum (Gibco, New York, NY, USA). The cells were cultured in a CO_2_ incubator at 37°C in 5% CO_2_ atmosphere at increased humidity.

The film samples were sterilized in an autoclave for 40 min at a temperature of 121 °C and a pressure of 1.5 atm. The films were cut into round pieces and placed in the wells of a 24-well culture plate; the suspension of the cells (25 × 10^3^) in a complete culture medium was added. The cells grown on cultural plastic were used for comparison.

MTT test was performed after 4 days of the cell cultivation; 100 μL of the working solution of MTT was added to each well, and the samples were incubated for 2 h. The resulting formazan crystals were extracted by adding 1 mL of DMSO into cells. Optical density of the obtained solution was registered at a wavelength of 570 nm with the use of a SPECTROstar Nano spectrophotometer. Optical density of this solution correlates with the amount of viable cells.

### 2.3. Study of the Influence of Electrostimulation on Vital Functions of Cells

**Figure 2:**
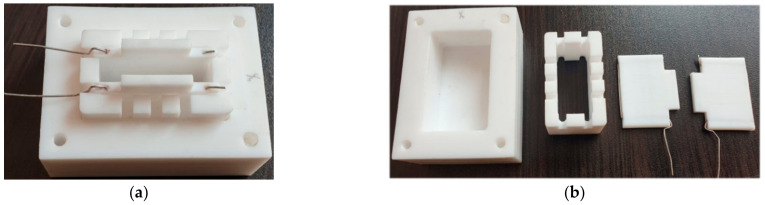
Assembled electrostimulation setup (**a**) and elements of the setup (**b**).

Design of the setup (Figure 2) used for investigation of the influence of electrostimulation on vital functions of the human dermal fibroblasts is described in [50]. 

The setup consisted of a Teflon bath, on the bottom of which was placed a flat film electrode and frame, fixing its position. The electrode was connected by a platinum wire of 1 mm in diameter to a power source. The setup was liquid-tight; it provided gas ex-change necessary for cellular process. 

The electrostimulation experiments were carried out with the use of an ELINS potentiostat-galvanostat (the operating current range: 10^−9^–2.0 A; the operating voltage range: 8 × 10^−6^–15.0 V; the rate of signal registration: 1580 points/s).

The electrostimulation experiments were conducted in the setup described above. The suspension of the cells (25 × 10^3^) in the complete culture medium was placed onto the surface of a sterilized electroconductive composite electrode film. The reference sample was a standard vial containing similar number of the dermal fibroblasts. Then the setup and the reference vial were placed into the CO_2_ incubator for 24 h. After 24 h, electrostimulation of the cells was performed for 4 h by continuous application of square-wave potential with a period of 60 s and an amplitude of ±100 mV to the film.

The choice of the external signal parameters was conditioned by limitations of work with the biological objects. On the one hand, the level of the electrode potential is almost similar to the membrane resting potentials (−70 mV) and the action potentials (+40 mV) of the living cells; on the other hand, it lies far from boundaries of the “stability window” of aqueous electrolyte (1.23 V). Applying the potential close to these boundaries may cause formation of free radicals, which are toxic to living organisms [39]. The frequency of potential change (0.33 Hz) is close to physiological parameters of an organism.

One hour after completion of electrostimulation, the fibroblasts from the setup and reference cell culture flasks were transferred to other flasks (surface area 12.5 cm^2^) and kept in the CO_2_ incubator until a monolayer was formed on the surface.

When the monolayer was formed, the cells were removed from vials and placed onto an 8-well plate of an RTCA iCELLIgence analyzer (5 × 10^3^ cells per well). The cell growth rate was monitored for 7 days by measuring electric impedance of the sensors located at the bottom of wells. The obtained value of the electric impedance corresponded to the value of cell index (CI) [51].

When there are no cells, or they are not attached to the film electrode, the CI value is equal to the background value (near zero). The CI values increase as the cells become attached to the electrodes.

**Figure 3:**
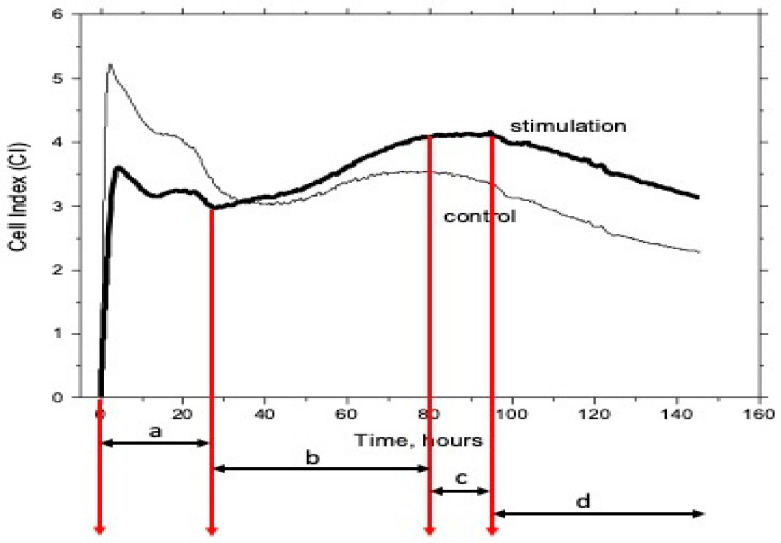
Life stages of the stimulated and reference cells on the cell index (CI) vs. time plot: a—adhesion, detachment, readhesion, spreading, b—proliferation, c—confluence plateau, d—initiation of apoptosis, apoptosis.

As a result, time dependences of the cell indices of the stimulated and reference cells were obtained. The plots were used to estimate dynamics of adhesion, proliferation of cells, and beginning of stationary phase of growth of the stimulated cells in comparison to those of the intact cells (Figure 3) [52].

## 3. Results and Discussion 

**Figure 4:**
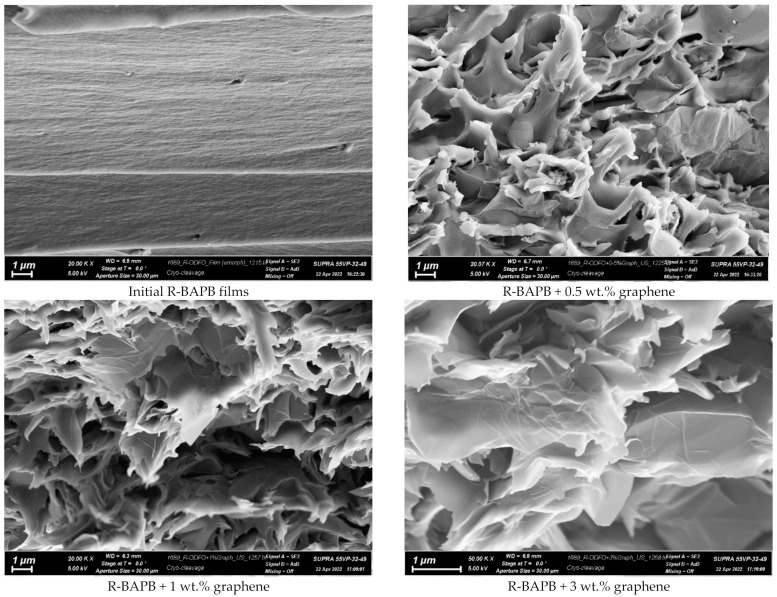
The SEM photographs of the original polyimide film R-BAPB and composites with graphene.

The results of the study of the structure of composite films based on R-BAPB containing graphene particles by the SEM method are shown in Figure 4.

It is shown that the initial film has a dense layered structure. The PI layers are oriented mainly in the plane of the film (Figure 4). Introduction of 0.5 wt.% graphene leads to a significant change in the structure of the composite (Figure 4). There is no orientation of the layers.

The composite films on the basis of aromatic PI and graphene should possess sufficient mechanical strength and deformation properties for application as bioelectrodes in cell technologies. The research demonstrated that the strength of the films containing 0.5–1.0 wt.% of graphene is equal to 75–85 MPa, and their tensile strain is equal to 35–45%. The films possessing these mechanical characteristics can be subjected to various technological manipulations both in air and in liquid media. An increase in graphene percentage is observed to lead to a decrease in strength and tensile strain, which is caused by the formation of a graphene percolation cluster.

### 3.1. Electrical Properties of the Graphene-Containing Films on the Basis of R-BAPB

**Figure 5:**
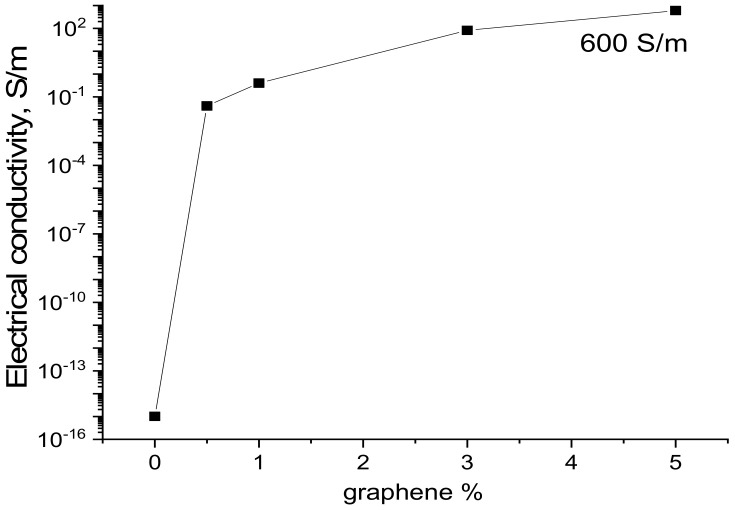
Dependence of conductivity of the R-BAPB-based composite films on graphene content.

Figure 5 presents the dependence of the conductivity of the composite films on graphene content. The R-BAPB film has low conductivity (10^−15^ S/m) and can be classed as a dielectric material, which agrees with the data reported in [53]. An increase in the graphene content in the composite results in an increase in conductivity. The percolation threshold of electric conductivity lies near 0.5 wt.% of graphene content. 

Graphene particles form a dimensionless conducting cluster; its conductivity is partially caused by the tunnel transition of electrons from one graphene particle to another. With increasing the graphene percentage in composites, electronic conductivity begins to prevail. 

**Figure 6:**
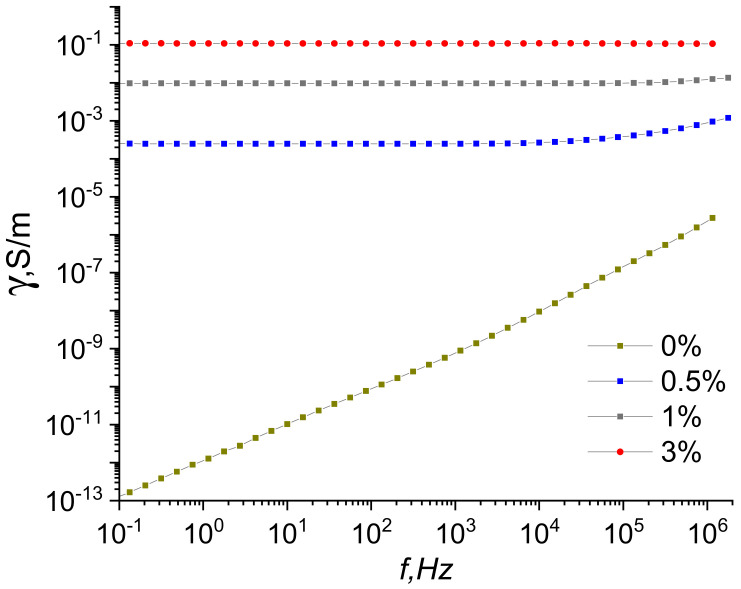
Frequency dependences of conductivity for composites containing 0, 0.5, 1.0, and 3.0 wt.% of graphene.

The dependences of the electric conductivity of the PI/graphene composite films on the frequency of the electric field are shown in Figure 6.

Figure 6 demonstrates that the real part of the conductivity of a pure PI film increases with the increasing frequency of the electric field, which is caused by the dielectric properties of this film. Introducing graphene nanoparticles considerably increases the conductivity of the composite films; its value virtually does not depend on frequency. This result indicates a high density of charge carriers, which are able to spread rapidly in the conducting graphene cluster due to the phase coincidence of the current and voltage at high frequencies. A certain discrepancy between conductivity measurement results for direct and alternating currents is caused by the use of different techniques. The direct current measurements were performed according to the four-electrode technique, when the current flows mainly in the near-surface areas. The alternating current measurements were carried out according to the two-electrode system; in this case, the current flows through the whole sample. Therefore, the surfaces of composite films have higher conductivity than their internal areas.

### 3.2. Volt-Ampere Characteristic of the Graphene-Containing R-BAPB Films

The study of the influence of the electric field on cell processes requires information about the value and type of the current flowing through the film placed into the measurement setup. The composite films were studied both in the dry state and in a physiological solution (0.9% aqueous solution of NaCl), i.e., under conditions approximated to the procedure of electrostimulation of the cells.

**Figure 7:**
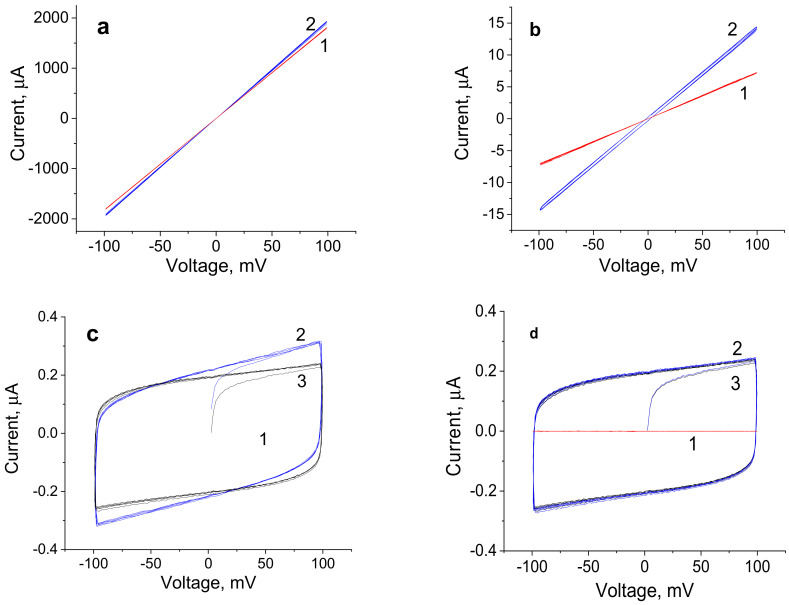
Voltage-current relationships of the R-BAPB films containing (**a**) 3.0 wt.%, (**b**) 1.0 wt.%, (**c**) 0.5 wt.% of graphene, and (**d**) pure polyimide film. 1-dry samples; 2-samples immersed in the electrolyte (NaCl 0.9%); (**c**) 3-electrodes of the cell with electrolyte (NaCl 0.9%) and without the film.

Figure 7 presents voltage-current relationships of the films containing 3.0, 1.0, 0.5, and 0 wt.% of graphene in the ±100 mV voltage range.

When the content of graphene is equal to 3.0 wt.%, the conductivity of the composite film reaches 100 S/m, and the conductivity of the film containing 0.5 wt.% is equal to 0.1 S/m; i.e., this value decreases by more than three orders of magnitude. This value of conductivity is one order of magnitude lower than the conductivity of the physiological solution. For dry samples (red lines), all voltage-current relationships are linear (Figure 7a-c, curves 1) and change exactly together with changes in potential. Therefore, these samples demonstrate electronic conductivity. For the sample containing 3.0 wt.% of graphene, the voltage-current relationship in the electrolyte solution (blue curve) is also linear and coincides with the similar dependence for the dry sample. This result indicates that ionic currents of the electrolyte are considerably lower than electronic currents and do not make a noticeable contribution to the conductivity of the sample. The current flows predominantly through the film containing the conducting graphene macrocluster.

When the graphene content decreases down to 1.0 wt.%, ionic currents begin to manifest themselves together with electronic currents, which is implied by the change in the slope of the curve and the beginning of the formation of a cycle at direct and reverse applied potentials. For the sample containing 1.0 wt.% of graphene, the contribution of the electronic component is approximately one order of magnitude higher than the ionic current of the electrolyte.

In the case of the sample containing 0.5 wt.% of graphene, the electronic current in the scaffold is lower than ionic currents in the electrolyte (Figure 7c). Here, the main current is ionic and flows through the electrolyte, as witnessed by the shape of the voltage-current relationship (curve reaching plateau, Figure 7c, curve 3). Under potential cycling, the capacitance component (which corresponds to electrochemical capacity) appears. The capacity of the sample (blue cycle) is somewhat higher than the capacity of the double electric layer of cell electrodes (black cycle), which indicates an increase in the area of the double electric layer in the presence of the film. This result agrees with the hypothesis about the structure of the composite: namely, graphene is embedded into the intermolecular space of the R-BAPB polyimide and thus causes the formation of pores available for the electrolyte ions. However, when the potential is applied to the sample containing 0.5 wt.% of graphene, the current flows mainly through the electrolyte, along the interface of polymer composite films, and not within the sample. Similarly, the current flows through the electrolyte after applying cyclic potential to the pure R-BAPB film (Figure 7d).

### 3.3. Analysis of Biocompatibility of the Composite Films

To study the biocompatibility of the films, human dermal fibroblasts were subjected to an MTT test after 4 days of cultivation on the surface of the composite films. The number of viable cells contacting the scaffolds was determined from the value of the optical absorption coefficient D of the solution containing (3-(4,5-dimethylthiazol-2-yl)-2,5-diphenyltetrazolium bromide) [54]. Optical density of the formazan solution correlates with the amount of viable cells.

**Figure 8:**
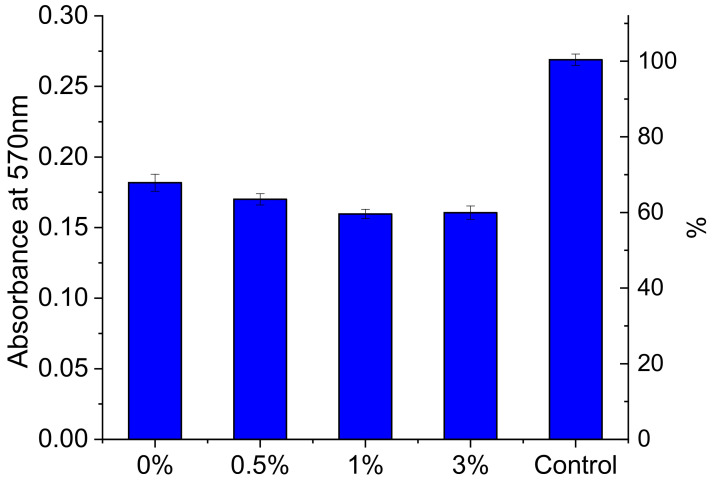
Results of MTT test for the graphene/R-BAPB composite films.

Figure 8 presents the results of the MTT test involving the cells grown on the surface of R-BAPB-based composite films containing graphene. The obtained data indicate that the studied composite films are generally biocompatible; human dermal fibroblasts adhered to their surface. At the same time, the value of the optical absorption coefficient D of the formazan solution measured at 570 nm is slightly lower for the composites (as compared to that of the pure film). An increase in the concentration of graphene in the composite from 1 to 3 wt.% does not cause a progressive decrease in the survival and proliferative activity of the cells when cultured on the composite films. This indicates an insignificant decrease in the number of viable cells on the composite films. From the results of the MTT test, it can be inferred that the R-BAPB/graphene composites can be used as flat bioelectrodes in the studies of the influence of the electric field on the adhesion, differentiation, and proliferation activity of stem and somatic cells.

### 3.4. Electrostimulation of Human Dermal Fibroblasts

All tissues of a living organism exhibit some degree of electrosensitivity. Their normal functions depend on electric signals delivered to cell membranes and can be modulated via exogenous electrical stimulation. Adhesion, proliferation, and differentiation of cells of an organ or a tissue can be controlled by changing parameters of electric impulses (voltage, amperage, frequency). 

In the present work, human dermal fibroblasts were used in the investigation of the influence of the electric field on cell processes. 

The choice of the type of electric signal was based on the assumption that the use of an alternating current would help avoid the negative influence of prolonged action of a direct current (polarization and imbalance of electric charge, accumulation of toxic side products, and electrochemical burns) [55].

Bioelectric currents with densities varying from 0.1 μA/cm^2^ to 1000 μA/cm^2^ constitute a critical factor in the launching and regulation of several important biological processes, including regeneration of damaged skin and soft tissues [56]. Therefore, this range of current densities was selected for studying the influence of electrostimulation on cell processes. In determining the physiological ranges of the voltage and frequency of electric signals, we used the data reported in [57]; the authors established the following optimal parameters for skin regeneration: voltage varying from 10 to 200 mV and the frequency not exceeding 1 Hz. 

The authors of [57,58] estimated the influence of the electric field on cell processes using the value of voltage gradient (i.e., electric field strength E). This characteristic seems more objective for estimating the influence of the electric field on the survivability, adhesion, and proliferation of the cells. A living cell is a complex system; its elements (plasmatic membrane, mitochondria, vacuoles, nucleus, etc.) are characterized by electric charges and potentials, their distribution both inside the cell and on its surface. Field strength as a spatial characteristic describes the interaction between the electric field of a setup and the external electric field more correctly. In the course of electrostimulation, field strength was calculated according to the substitution scheme from the value of applied voltage and geometry of the electric cell.

**Figure 9:**
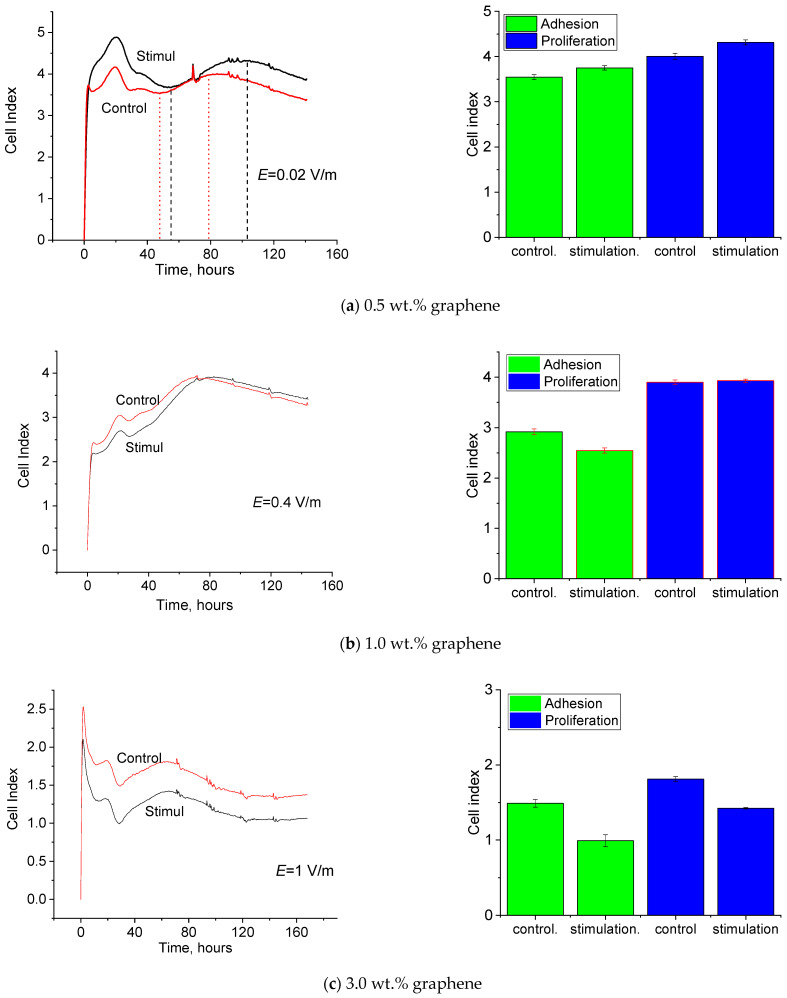

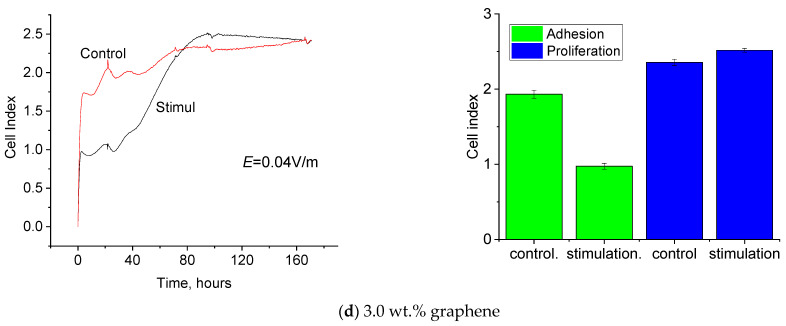
Dependences of the value of cell index (CI) on the time of cultivation of human fibroblasts on the R-BAPB films containing 0.5, 1.0, and 3.0 wt.% of graphene (**a**–**d**) in electric field with strength E = 0.02; 0.4; 1.0 and 0.04 V/m.

Figure 9 presents the time dependences of the cell index value (CI); the on-line analysis was performed using the RTCA iCELLIgence system. These curves allow one to estimate the dynamics of the adhesion and proliferation of the human dermal fibroblasts and CI at the moments of time corresponding to efficient adhesion and the maximum proliferation of the subsequent passages after electrostimulation on the composite films containing 0.5, 1.0, and 3.0 wt.% of graphene.

The CI values obtained for the cells stimulated on the composite film containing 0.5 wt.% of graphene at amperage ±0.4 μA and field strength 0.02 V/m at the time points corresponding to efficient adhesion and maximum proliferation are higher than the CI of the intact cells (Figure 9a). As shown above, this sample demonstrates ionic conductivity in the electrolyte. Therefore, in the course of electrostimulation of the cells grown on the film surface, the electric signal spreads predominantly in the culture medium but is localized in the near-surface layer of the medium, which improves the reception of the electric signal by the cells [39]. 

The CI values for the cells stimulated on the composite film containing 1 wt.% of graphene at amperage ±20 μA and field strength 0.4 V/m at the time points corresponding to efficient adhesion and maximum proliferation are virtually similar to those of the intact cells (Figure 9b). It was shown that this sample demonstrated electronic conductivity after immersion into the electrolyte. In the course of electrostimulation of the cells grown on the film surface, the electric signal spreads predominantly within the material. The electric field strength is too high (lies above the physiological range).

The CI values for the cells stimulated on the composite film containing 3 wt.% of graphene at amperage ±2500 μA and field strength 1 V/m at the time points of efficient adhesion and maximum proliferation are lower than the corresponding parameters of the intact cells (Figure 9c). When this sample was immersed in the electrolyte, the electric signal spread at the expense of the electronic conductivity of the material. Amperage and electric field strength are too high and do not fit into physiological ranges. To create more favorable conditions for electrostimulation, the electric field strength was decreased to 0.04 V/m.

The CI value for the cells stimulated on the composite film containing 3 wt.% of graphene at amperage ±100 μA and field strength 0.04 V/m at the time point corresponding to efficient adhesion is lower than that for intact cells. However, at the time point corresponding to efficient proliferation, CI increases significantly and exceeds the value for intact cells (Figure 9c). As shown above, for this sample immersed in the electrolyte, the electric signal spreads at the expense of the electronic conductivity of the material and (to a lesser degree) ionic conductivity of the medium. The values of amperage and electric field strength lie within physiological ranges.

## 4. Conclusions

The conducting composite films on the basis of R-BAPB filled with graphene were prepared. The incorporation of graphene into a polyimide matrix had a significant influence on the structure, electrical conductivity, and mechanical properties of polyimide. The values of specific conductivity of the composite films increased from 10^−15^ to 10^2^ S/m with increasing graphene content (from 0 to 5.0 wt.%).

Analysis of the voltage-current relationships of the composite films obtained in the dry state and in the physiological solutions in the voltage range ±100 mV showed that in the sample containing 0.5 wt.% of graphene, ionic conductivity (caused by electrolyte ions) prevails. In the case of the film containing 3.0 wt.% of graphene, electronic conductivity is predominant; the current flows on the surface along the film.

The results of the MTT test imply that the R-BAPB/graphene composite films are biocompatible and thus can be used as flat bioelectrodes in the studies of the influence of the electric field on the adhesion, proliferation activity, and differentiation of stem and somatic cells and as interfaces in medical devices.

The studies of the dependence of the cell index (CI) on cultivation time demonstrated that the films containing 0.5 wt.% of graphene (which possess mostly ionic conductivity) can be efficient as electrodes in the investigation of the influence of the electric field on the adhesion and proliferation activity of the human dermal fibroblasts.

It was demonstrated that an electric field with strength E = 0.02–0.04 V/m applied to the R-BAPB films containing 0.5–3.0 wt.% of the graphene nanoparticles activated cell processes (adhesion, spreading, proliferation). This result can be used to treat the skin.

## Data Availability

Not applicable.
